# Poly[bis­(μ_2_-4,4′-bipyridine)­bis­(3-nitro­benzoato)nickel(II)]

**DOI:** 10.1107/S1600536810050117

**Published:** 2010-12-11

**Authors:** Shih-Chen Hsu, Sheng-Han Lo, Ching-Che Kao, Chia-Her Lin

**Affiliations:** a Department of Chemistry, Chung-Yuan Christian University, Chung-Li 320, Taiwan

## Abstract

The crystal structure of the title complex, [Ni(C_7_H_4_NO_4_)_2_(C_10_H_8_N_2_)_2_]_*n*_, exhibits a two-dimensional network, which is built up from slightly distorted NiN_4_O_2_ polyhedra (2 symmetry), bipyridine ligands, and carboxyl­ate anions. The Ni^II^ atoms are six-coordinated by two O atoms of two monodentate carboxyl­ate anions and four N atoms from bipyridine ligands and are connected into layers by the 4,4′-bipyridine ligands.

## Related literature

For background to the hydro­thermal synthesis of coordination polymers with organic ligands, see: Kitagawa *et al.* (2004 ▶); Long & Yaghi (2009 ▶). For related structures, see: Chiang *et al.* (2009 ▶).
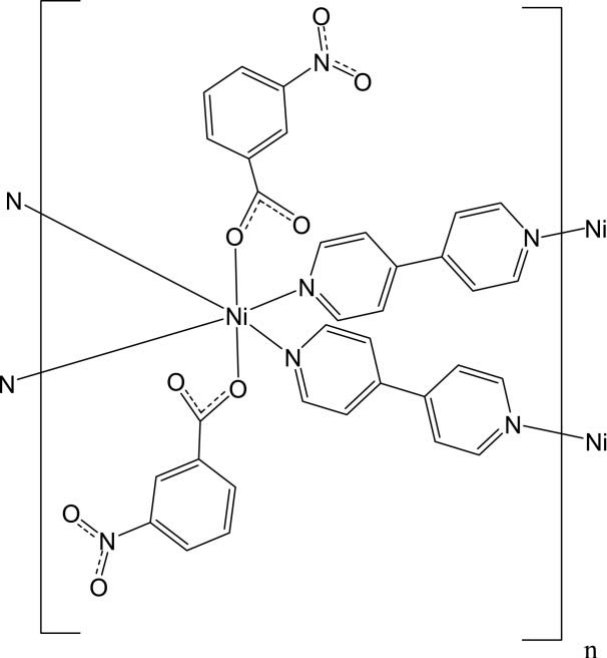


## Experimental

### 

#### Crystal data

[Ni(C_7_H_4_NO_4_)_2_(C_10_H_8_N_2_)_2_]
*M* *_r_* = 703.28
Monoclinic, 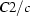

*a* = 18.1237 (10) Å
*b* = 11.3663 (6) Å
*c* = 15.0119 (8) Å
β = 95.439 (2)°
*V* = 3078.5 (3) Å^3^
*Z* = 4
Mo *K*α radiation
μ = 0.70 mm^−1^
*T* = 295 K
0.45 × 0.30 × 0.10 mm

#### Data collection

Bruker APEXII CCD diffractometer
Absorption correction: multi-scan (*SADABS*; Bruker, 2000 ▶) *T*_min_ = 0.745, *T*_max_ = 0.934
13263 measured reflections
3836 independent reflections
3470 reflections with *I* > 2σ(*I*)
*R*_int_ = 0.023

#### Refinement

*R*[*F* ^2^ > 2σ(*F* ^2^)] = 0.035
*wR*(*F* ^2^) = 0.101
*S* = 1.03
3836 reflections
224 parameters
H-atom parameters constrained
Δρ_max_ = 0.71 e Å^−3^
Δρ_min_ = −0.53 e Å^−3^

### 

Data collection: *APEX2* (Bruker, 2010 ▶); cell refinement: *SAINT* (Bruker, 2010 ▶); data reduction: *SAINT*; program(s) used to solve structure: *SHELXS97* (Sheldrick, 2008 ▶); program(s) used to refine structure: *SHELXL97* (Sheldrick, 2008 ▶); molecular graphics: *DIAMOND* (Brandenburg, 2010 ▶); software used to prepare material for publication: *SHELXTL* (Sheldrick, 2008 ▶).

## Supplementary Material

Crystal structure: contains datablocks I, global. DOI: 10.1107/S1600536810050117/jh2232sup1.cif

Structure factors: contains datablocks I. DOI: 10.1107/S1600536810050117/jh2232Isup2.hkl

Additional supplementary materials:  crystallographic information; 3D view; checkCIF report

## Figures and Tables

**Fig. 1.**
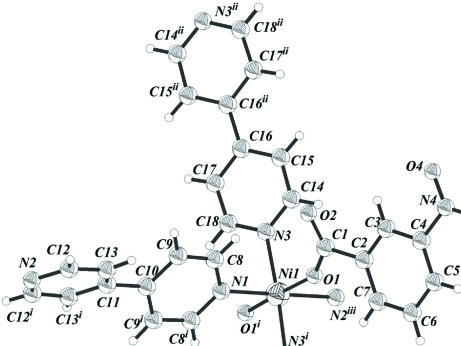
Part of polymeric structure of the title compound, showing 50% probability displacement ellipsoids. [symmetry codes: (i) -x + 1, y, -z + 5/2; (ii) -x + 1/2, -y + 5/2, -z + 2; (iii) x, y - 1, z].

**Fig. 2.**
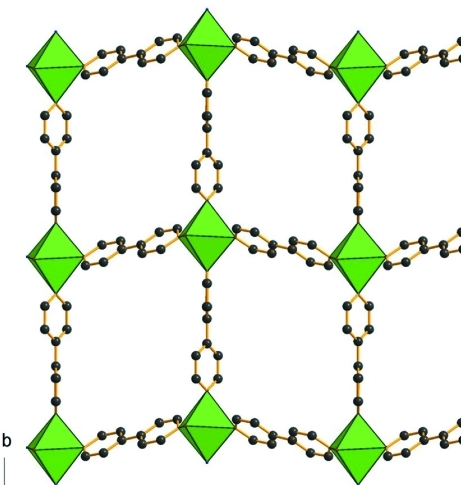
Crystal structure of the title compound showing the two-dimensional layers. The H atoms are omitted for clarity.

## supplementary crystallographic information

### Comment

The synthesis of metal coordination polymers has been a subject of intense research due to their interesting structural chemistry and potential applications in gas storage, separation, catalysis, magnetism, luminescence, and drug delivery (Kitagawa, *et al.*, 2004). Here we report the synthesis of title complex with a two-dimensional structure which was contained nickel and mixed 4,4'-bipyridine and 3-nitrobenzate ligands.

The crystal structure analysis reveals that the title complex possesses similar two-dimensional layer structure. The octahedral metal ions are coordinated by four nitrogen atoms and two oxygen atoms which belonged to the four bpy ligands, and two NB ligands (Fig. 1). The average bond lengths of Ni—O are 2.058 (1) Å and the Ni—N are 2.128 (2) Å which are falling in the expected normal range. Each metal center was linked adjacent metal centers by four bpy ligands, resulting in a two-dimensional neutral rectangular grid in the *bc* plane with a (4,4)-net (Fig. 2). The neighboring layers interact through π-π interactions between the benzene rings of NB ligands (3.55 Å) and form a mimic three-dimensional framework. In generally, the title complex is an analogous of our precious reported cobalt compound (Chiang, *et al.*, 2009).

### Experimental

Hydrothermal reactions were carried out at 453 K for 3 d in a Teflon-lined acid digestion bomb with an internal volume of 23 ml followed by slow cooling at 6 K/h to room temperature. A single-phase product consisting of blue crystals were obtained from a mixture of 4,4'-bipyridine (C_10_H_8_N_2_, 0.0781 g, 0.5 mmol), 3-nitrobenzoic acid (C_7_H_5_NO_4_, 0.0836 g, 0.5 mmol), Ni(NO_3_)_2_?6H_2_O (0.1454 g, 0.5 mmol), and H_2_O (12.0 ml), NH_4_OH (0.1 ml).

### Refinement

H atoms were constrained to ideal geometries, with C—H = 0.93 Å and *U*_iso_(H) = 1.2*U*_eq_(C).

### Crystal data

**Table:**

[Ni(C_7_H_4_NO_4_)_2_(C_10_H_8_N_2_)_2_]	*F*(000) = 1448
*M**_r_* = 703.28	*D*_x_ = 1.517 Mg m^−^^3^
Monoclinic, *C*2/*c*	Mo *K*α radiation, λ = 0.71073 Å
Hall symbol: -C 2yc	Cell parameters from 7469 reflections
*a* = 18.1237 (10) Å	θ = 2.3–28.4°
*b* = 11.3663 (6) Å	µ = 0.70 mm^−^^1^
*c* = 15.0119 (8) Å	*T* = 295 K
β = 95.439 (2)°	Columnar, blue
*V* = 3078.5 (3) Å^3^	0.45 × 0.30 × 0.10 mm
*Z* = 4	

### Data collection

**Table:**

Bruker APEXII CCD diffractometer	3836 independent reflections
Radiation source: fine-focus sealed tube	3470 reflections with *I* > 2σ(*I*)
graphite	*R*_int_ = 0.023
Detector resolution: 8.3333 pixels mm^-1^	θ_max_ = 28.4°, θ_min_ = 2.1°
φ and ω scans	*h* = −24→24
Absorption correction: multi-scan (*SADABS*; Bruker, 2000)	*k* = −10→15
*T*_min_ = 0.745, *T*_max_ = 0.934	*l* = −20→18
13263 measured reflections	

### Refinement

**Table:**

Refinement on *F*^2^	Primary atom site location: structure-invariant direct methods
Least-squares matrix: full	Secondary atom site location: difference Fourier map
*R*[*F*^2^ > 2σ(*F*^2^)] = 0.035	Hydrogen site location: inferred from neighbouring sites
*wR*(*F*^2^) = 0.101	H-atom parameters constrained
*S* = 1.03	*w* = 1/[σ^2^(*F*_o_^2^) + (0.055*P*)^2^ + 3.5308*P*] where *P* = (*F*_o_^2^ + 2*F*_c_^2^)/3
3836 reflections	(Δ/σ)_max_ = 0.002
224 parameters	Δρ_max_ = 0.71 e Å^−^^3^
0 restraints	Δρ_min_ = −0.53 e Å^−^^3^

### Special details

**Table:**

Geometry. All e.s.d.'s (except the e.s.d. in the dihedral angle between two l.s. planes) are estimated using the full covariance matrix. The cell e.s.d.'s are taken into account individually in the estimation of e.s.d.'s in distances, angles and torsion angles; correlations between e.s.d.'s in cell parameters are only used when they are defined by crystal symmetry. An approximate (isotropic) treatment of cell e.s.d.'s is used for estimating e.s.d.'s involving l.s. planes.
Refinement. Refinement of *F*^2^ against ALL reflections. The weighted *R*-factor *wR* and goodness of fit *S* are based on *F*^2^, conventional *R*-factors *R* are based on *F*, with *F* set to zero for negative *F*^2^. The threshold expression of *F*^2^ > σ(*F*^2^) is used only for calculating *R*-factors(gt) *etc*. and is not relevant to the choice of reflections for refinement. *R*-factors based on *F*^2^ are statistically about twice as large as those based on *F*, and *R*- factors based on ALL data will be even larger.

### Fractional atomic coordinates and isotropic or equivalent isotropic displacement parameters (Å^2^)

**Table:**

	*x*	*y*	*z*	*U*_iso_*/*U*_eq_	
Ni1	0.5000	1.19295 (2)	1.2500	0.02130 (10)	
O1	0.57303 (7)	1.18647 (10)	1.15316 (8)	0.0311 (3)	
N1	0.5000	1.37958 (17)	1.2500	0.0274 (4)	
C1	0.58736 (10)	1.20357 (14)	1.07335 (12)	0.0298 (3)	
O2	0.54917 (9)	1.25698 (16)	1.01439 (10)	0.0515 (4)	
C2	0.66083 (10)	1.15107 (15)	1.05145 (12)	0.0318 (3)	
N2	0.5000	2.00284 (16)	1.2500	0.0264 (4)	
O3	0.81171 (16)	1.0200 (3)	0.83539 (19)	0.1131 (10)	
N3	0.40568 (8)	1.19913 (11)	1.15701 (9)	0.0261 (3)	
C3	0.67500 (11)	1.13723 (17)	0.96282 (13)	0.0387 (4)	
H3A	0.6403	1.1601	0.9165	0.046*	
O4	0.70979 (17)	1.1035 (3)	0.79302 (15)	0.1014 (9)	
N4	0.75517 (16)	1.0686 (2)	0.85108 (18)	0.0693 (7)	
C4	0.74173 (13)	1.08876 (19)	0.94515 (15)	0.0474 (5)	
C5	0.79625 (13)	1.0578 (2)	1.01120 (18)	0.0529 (6)	
H5A	0.8413	1.0273	0.9969	0.063*	
C6	0.78225 (12)	1.0733 (2)	1.09890 (17)	0.0500 (5)	
H6A	0.8184	1.0544	1.1448	0.060*	
C7	0.71441 (11)	1.11721 (17)	1.11888 (14)	0.0395 (4)	
H7A	0.7046	1.1241	1.1784	0.047*	
C8	0.49914 (10)	1.44061 (15)	1.17354 (11)	0.0323 (4)	
H8A	0.4989	1.3993	1.1201	0.039*	
C9	0.49863 (11)	1.56217 (15)	1.17042 (12)	0.0343 (4)	
H9A	0.4974	1.6013	1.1159	0.041*	
C10	0.5000	1.6253 (2)	1.2500	0.0289 (5)	
C11	0.5000	1.75560 (19)	1.2500	0.0292 (5)	
C12	0.53631 (16)	1.94065 (18)	1.19430 (19)	0.0637 (8)	
H12A	0.5634	1.9812	1.1546	0.076*	
C13	0.53672 (18)	1.81906 (18)	1.1911 (2)	0.0685 (9)	
H13A	0.5621	1.7805	1.1486	0.082*	
C14	0.40194 (9)	1.15368 (17)	1.07508 (12)	0.0320 (4)	
H14A	0.4413	1.1079	1.0595	0.038*	
C15	0.34223 (9)	1.17121 (17)	1.01177 (12)	0.0333 (4)	
H15A	0.3423	1.1383	0.9551	0.040*	
C16	0.28214 (8)	1.23809 (14)	1.03306 (10)	0.0258 (3)	
C17	0.28557 (10)	1.28207 (17)	1.11967 (12)	0.0337 (4)	
H17A	0.2461	1.3252	1.1381	0.040*	
C18	0.34745 (10)	1.26177 (17)	1.17822 (11)	0.0331 (4)	
H18A	0.3488	1.2933	1.2355	0.040*	

### Atomic displacement parameters (Å^2^)

**Table:**

	*U*^11^	*U*^22^	*U*^33^	*U*^12^	*U*^13^	*U*^23^
Ni1	0.02510 (15)	0.01629 (15)	0.02124 (15)	0.000	−0.00447 (10)	0.000
O1	0.0359 (6)	0.0310 (6)	0.0264 (6)	−0.0015 (5)	0.0027 (5)	0.0002 (5)
N1	0.0350 (10)	0.0183 (8)	0.0277 (9)	0.000	−0.0025 (7)	0.000
C1	0.0353 (8)	0.0257 (8)	0.0280 (8)	−0.0033 (6)	0.0005 (6)	0.0002 (6)
O2	0.0540 (9)	0.0647 (10)	0.0352 (7)	0.0153 (8)	0.0006 (6)	0.0132 (7)
C2	0.0397 (9)	0.0245 (8)	0.0318 (8)	−0.0039 (7)	0.0063 (7)	0.0018 (7)
N2	0.0301 (9)	0.0181 (8)	0.0300 (9)	0.000	−0.0026 (7)	0.000
O3	0.116 (2)	0.138 (2)	0.0964 (18)	0.0219 (18)	0.0682 (17)	−0.0171 (17)
N3	0.0267 (6)	0.0246 (7)	0.0255 (6)	0.0016 (5)	−0.0053 (5)	−0.0006 (5)
C3	0.0510 (11)	0.0325 (9)	0.0339 (9)	−0.0080 (8)	0.0110 (8)	0.0009 (7)
O4	0.124 (2)	0.142 (2)	0.0431 (11)	0.0011 (18)	0.0311 (13)	−0.0084 (13)
N4	0.0897 (17)	0.0634 (14)	0.0620 (14)	−0.0163 (13)	0.0458 (14)	−0.0110 (11)
C4	0.0611 (13)	0.0358 (10)	0.0494 (12)	−0.0121 (9)	0.0271 (10)	−0.0027 (9)
C5	0.0479 (11)	0.0395 (11)	0.0751 (16)	0.0005 (9)	0.0260 (11)	0.0024 (11)
C6	0.0432 (11)	0.0449 (12)	0.0619 (14)	0.0054 (9)	0.0052 (10)	0.0076 (10)
C7	0.0441 (10)	0.0355 (9)	0.0390 (10)	0.0014 (8)	0.0054 (8)	0.0047 (8)
C8	0.0471 (9)	0.0209 (8)	0.0282 (8)	0.0022 (7)	−0.0007 (7)	−0.0016 (6)
C9	0.0517 (10)	0.0207 (8)	0.0302 (8)	0.0017 (7)	0.0020 (7)	0.0035 (6)
C10	0.0360 (11)	0.0164 (10)	0.0339 (12)	0.000	0.0009 (9)	0.000
C11	0.0380 (12)	0.0169 (10)	0.0321 (11)	0.000	0.0005 (9)	0.000
C12	0.0924 (19)	0.0214 (9)	0.0867 (19)	0.0000 (10)	0.0572 (17)	0.0040 (10)
C13	0.104 (2)	0.0216 (10)	0.090 (2)	0.0037 (11)	0.0647 (19)	−0.0007 (10)
C14	0.0261 (7)	0.0354 (9)	0.0330 (8)	0.0052 (6)	−0.0053 (6)	−0.0092 (7)
C15	0.0285 (8)	0.0419 (10)	0.0279 (8)	0.0051 (7)	−0.0053 (6)	−0.0105 (7)
C16	0.0253 (7)	0.0260 (8)	0.0250 (7)	−0.0001 (6)	−0.0027 (6)	0.0009 (6)
C17	0.0324 (8)	0.0425 (10)	0.0252 (8)	0.0131 (7)	−0.0027 (6)	−0.0022 (7)
C18	0.0359 (8)	0.0396 (9)	0.0224 (7)	0.0104 (7)	−0.0044 (6)	−0.0036 (7)

### Geometric parameters (Å, °)

**Table:**

Ni1—O1	2.0580 (12)	C5—H5A	0.9300
Ni1—O1^i^	2.0580 (12)	C6—C7	1.386 (3)
Ni1—N3	2.1031 (13)	C6—H6A	0.9300
Ni1—N3^i^	2.1031 (13)	C7—H7A	0.9300
Ni1—N1	2.1212 (19)	C8—C9	1.382 (2)
Ni1—N2^ii^	2.1609 (19)	C8—H8A	0.9300
O1—C1	1.265 (2)	C9—C10	1.391 (2)
N1—C8	1.340 (2)	C9—H9A	0.9300
N1—C8^i^	1.340 (2)	C10—C9^i^	1.391 (2)
C1—O2	1.231 (2)	C10—C11	1.482 (3)
C1—C2	1.523 (2)	C11—C13	1.363 (3)
C2—C7	1.389 (3)	C11—C13^i^	1.363 (3)
C2—C3	1.388 (2)	C12—C13	1.383 (3)
N2—C12	1.318 (2)	C12—H12A	0.9300
N2—C12^i^	1.318 (2)	C13—H13A	0.9300
N2—Ni1^iii^	2.1610 (19)	C14—C15	1.385 (2)
O3—N4	1.207 (3)	C14—H14A	0.9300
N3—C14	1.330 (2)	C15—C16	1.390 (2)
N3—C18	1.336 (2)	C15—H15A	0.9300
C3—C4	1.377 (3)	C16—C17	1.389 (2)
C3—H3A	0.9300	C16—C16^iv^	1.482 (3)
O4—N4	1.207 (4)	C17—C18	1.377 (2)
N4—C4	1.474 (3)	C17—H17A	0.9300
C4—C5	1.377 (4)	C18—H18A	0.9300
C5—C6	1.375 (4)		
			
O1—Ni1—O1^i^	175.90 (7)	C6—C5—H5A	120.9
O1—Ni1—N3	93.96 (5)	C4—C5—H5A	120.9
O1^i^—Ni1—N3	86.18 (5)	C5—C6—C7	120.1 (2)
O1—Ni1—N3^i^	86.18 (5)	C5—C6—H6A	120.0
O1^i^—Ni1—N3^i^	93.96 (5)	C7—C6—H6A	120.0
N3—Ni1—N3^i^	176.17 (7)	C6—C7—C2	121.0 (2)
O1—Ni1—N1	92.05 (3)	C6—C7—H7A	119.5
O1^i^—Ni1—N1	92.05 (3)	C2—C7—H7A	119.5
N3—Ni1—N1	88.09 (4)	N1—C8—C9	123.09 (16)
N3^i^—Ni1—N1	88.09 (4)	N1—C8—H8A	118.5
O1—Ni1—N2^ii^	87.95 (3)	C9—C8—H8A	118.5
O1^i^—Ni1—N2^ii^	87.95 (3)	C8—C9—C10	119.10 (16)
N3—Ni1—N2^ii^	91.91 (4)	C8—C9—H9A	120.4
N3^i^—Ni1—N2^ii^	91.91 (4)	C10—C9—H9A	120.4
N1—Ni1—N2^ii^	180.000 (1)	C9—C10—C9^i^	118.0 (2)
C1—O1—Ni1	150.13 (12)	C9—C10—C11	121.02 (10)
C8—N1—C8^i^	117.6 (2)	C9^i^—C10—C11	121.01 (10)
C8—N1—Ni1	121.18 (10)	C13—C11—C13^i^	116.1 (2)
C8^i^—N1—Ni1	121.18 (10)	C13—C11—C10	121.95 (12)
O2—C1—O1	127.24 (18)	C13^i^—C11—C10	121.96 (12)
O2—C1—C2	118.78 (16)	N2—C12—C13	124.3 (2)
O1—C1—C2	113.98 (15)	N2—C12—H12A	117.9
C7—C2—C3	119.16 (18)	C13—C12—H12A	117.9
C7—C2—C1	121.09 (16)	C11—C13—C12	120.1 (2)
C3—C2—C1	119.76 (17)	C11—C13—H13A	119.9
C12—N2—C12^i^	115.1 (2)	C12—C13—H13A	119.9
C12—N2—Ni1^iii^	122.44 (11)	N3—C14—C15	123.14 (16)
C12^i^—N2—Ni1^iii^	122.44 (11)	N3—C14—H14A	118.4
C14—N3—C18	117.08 (14)	C15—C14—H14A	118.4
C14—N3—Ni1	124.60 (11)	C14—C15—C16	119.94 (16)
C18—N3—Ni1	118.05 (11)	C14—C15—H15A	120.0
C4—C3—C2	118.4 (2)	C16—C15—H15A	120.0
C4—C3—H3A	120.8	C17—C16—C15	116.46 (14)
C2—C3—H3A	120.8	C17—C16—C16^iv^	121.59 (18)
O3—N4—O4	122.9 (3)	C15—C16—C16^iv^	121.95 (18)
O3—N4—C4	118.6 (3)	C18—C17—C16	119.91 (16)
O4—N4—C4	118.5 (2)	C18—C17—H17A	120.0
C3—C4—C5	123.1 (2)	C16—C17—H17A	120.0
C3—C4—N4	118.3 (2)	N3—C18—C17	123.43 (16)
C5—C4—N4	118.5 (2)	N3—C18—H18A	118.3
C6—C5—C4	118.1 (2)	C17—C18—H18A	118.3

Symmetry codes: (i) −*x*+1, *y*, −*z*+5/2; (ii) *x*, *y*−1, *z*; (iii) *x*, *y*+1, *z*; (iv) −*x*+1/2, −*y*+5/2, −*z*+2.
